# Early GLP-1 Agonist Use and Cancer Risk in Type 2 Diabetes: A Real-World Data Cohort Study

**DOI:** 10.32604/or.2025.072875

**Published:** 2025-12-30

**Authors:** Cheng-Hsun Chuang, Ping-Kun Tsai, Shih-Wen Kao, Yu-Hsun Wang, Chao-Bin Yeh

**Affiliations:** 1 Institute of Medicine, Chung Shan Medical University, Taichung, 402, Taiwan; 2 Department of Emergency Medicine, School of Medicine, Chung Shan Medical University, Taichung, 402, Taiwan; 3 Department of Emergency Medicine, Chung Shan Medical University Hospital, Taichung, 402, Taiwan; 4 Department of Internal Medicine, Zuoying Armed Forces General Hospital, Kaohsiung, 813, Taiwan; 5 Department of Emergency Medicine, Tri-Service General Hospital, National Defense Medical Center, Taipei, 114, Taiwan; 6 Graduate Institute of Aerospace and Undersea Medicine, College of Biomedical Sciences, National Defense Medical University, Taipei, 114, Taiwan; 7 Department of Orthopedic Surgery, Chung Shan Medical University Hospital, Taichung, 402, Taiwan; 8 School of Medicine, Chung Shan Medical University, Taichung, 402, Taiwan; 9 Department of Medical Research, Chung Shan Medical University Hospital, Taichung, 402, Taiwan

**Keywords:** Glucagon-like peptide-1 (GLP-1) receptor agonists, type 2 diabetes mellitus, cancer risk, obesity, cohort study

## Abstract

**Background:**

To determine whether initiating a glucagon-like peptide-1 receptor agonist (GLP-1 RA) within 3 months of type 2 diabetes (T2DM) diagnosis alters the subsequent risk of overall and site-specific cancer and whether this association differs by baseline body-mass index (BMI).

**Methods:**

This retrospective cohort study used electronic health records from the TriNetX U.S. research network. Adults aged 20 years or older diagnosed with T2DM between 2016 and 2024 were included if they received any hypoglycemic agents within 3 months before and after diagnosis. Following 1:1 propensity score matching, both the GLP-1 RA user and non-user groups included 183,264 patients. The study outcome was defined as a diagnosis of malignant neoplasms. Hazard ratios (HRs) for overall and site-specific cancer risk were estimated using Cox proportional hazards models. Kaplan–Meier analysis and stratified analysis by BMI were performed.

**Results:**

Early GLP-1 RA use demonstrated a modest but significant association with reduced overall cancer risk (HR 0.93; 95% CI: 0.90–0.96). Reduced risks were noted for cancers of the digestive (HR 0.81), respiratory (HR 0.66), and female genital (HR 0.87) systems. In stratified analysis, benefits were more pronounced in patients with BMI ≥ 30, particularly for pancreatic and colorectal cancers.

**Conclusion:**

Early initiation of GLP-1 receptor agonists in patients with diagnosed T2DM was associated with a modest reduction in overall cancer risk, particularly among individuals with obesity. These findings highlight the dual metabolic and oncologic value of prompt GLP-1 RA therapy.

## 1 Introduction

Glucagon-like peptide-1 receptor agonist (GLP-1 RA) is a class of incretin-based therapies widely used in the management of type 2 diabetes mellitus (T2DM). By mimicking endogenous GLP-1, these agents enhance glucose-dependent insulin secretion, suppress glucagon release, delay gastric emptying, and promote satiety [1,2]. GLP-1 effects are mediated through the GLP-1 receptor (GLP-1R), a G-protein coupled receptor expressed not only in pancreatic β-cells but also in the cardiovascular system, adipose tissue, and central nervous system. Activation of GLP-1R supports glucose homeostasis while also influencing weight regulation, appetite, and cardiovascular function, underscoring its physiological relevance beyond glycemic control [3]. Upon receptor activation, GLP-1 elevates intracellular cAMP, which activates protein kinase A (PKA) and EPAC (Exchange Protein directly Activated by cAMP), thereby facilitating insulin secretion and attenuating glucagon release. In addition, GLP-1 signaling engages the PI3K/AKT pathway, which is essential for β-cell survival and function, and can also activate MAPK/ERK cascades involved in cell growth and proliferation [3,4]. By addressing both hyperglycemia and excess body weight, GLP-1 RA have become a cornerstone in the treatment of patients with T2DM, particularly those with concurrent obesity [2,5]. Beyond glycemic control, GLP-1 RA have demonstrated cardiovascular benefits in numerous large-scale randomized controlled trials. [6] Agents such as liraglutide, semaglutide, and dulaglutide have been associated with reductions in major adverse cardiovascular events (MACE) and cardiovascular mortality [7–9]. These findings have expanded the therapeutic role of GLP-1 RA to include cardiovascular risk reduction in patients with T2DM.

Despite these favorable outcomes, concerns remain regarding the long-term safety of GLP-1 RA, particularly their potential oncogenic effects. Preclinical rodent studies have shown that GLP-1 receptor activation can stimulate thyroid C-cell proliferation, raising concerns about the risk of medullary thyroid carcinoma (MTC). Supporting this theoretical risk, some epidemiological studies have suggested a possible association between prolonged GLP-1 RA exposure and an increased risk of thyroid malignancies, including MTC [10,11]. In contrast, a recent large multisite cohort study across six countries found no significant association between GLP-1 RA use and thyroid cancer compared with DPP-4 inhibitors (HR 0.81; 95% CI, 0.59–1.12), and no dose–response relationship was observed [12]. In addition to thyroid cancer, observational and pharmacovigilance studies have investigated potential associations between GLP-1 RA use and other malignancies—including pancreatic, colorectal, and breast cancers—with inconsistent results [13–15]. Some studies suggest protective effects, while others raise concerns about increased risk. However, many of these findings are complicated by population heterogeneity, underlying comorbidities, and differences in study design.

Given the growing use of GLP-1 RA in both diabetic and non-diabetic populations (e.g., for obesity management), it is important to critically evaluate their potential association with cancer risk. Importantly, there has been a shift in clinical practice toward initiating these agents earlier in the T2DM disease course. Previously, GLP-1 RA were typically introduced several years after diagnosis; now, they are often prescribed within months of diagnosis. However, the oncologic implications of such early use remain poorly understood. Accordingly, the present study seeks to explore the association between early initiation of GLP-1 RA therapy—defined as within three months of T2DM diagnosis—and the subsequent risk of developing cancer, with particular focus on site-specific malignancies potentially influenced by GLP-1 receptor signaling. We also conducted stratified analyses by body mass index (BMI) to assess whether the relationship between GLP-1 RA use and cancer risk differs according to obesity status.

## 2 Materials and Methods

### 2.1 Data Collection

This retrospective cohort study leverages electronic health records from TriNetX, which includes data from approximately 126 million patients within the United States collaborative network. Data were extracted and analyzed on 18 April 2025, and the database is broadly representative of patients receiving care in participating institutions across the U.S. The anonymized data, collected from a variety of healthcare institutions such as hospitals, primary care providers, and specialty providers, encompasses demographic information, diagnoses (using the International Classification of Diseases, Tenth Revision, Clinical Modification; ICD-10-CM), medications (standardized by RxNorm for prescriptions and Vaccine Administered Code; CVX for vaccines), procedures (using the International Classification of Diseases, Tenth Revision, Procedure Coding System; ICD-10-PCS, and Current Procedural Terminology; CPT), and laboratory results (using Logical Observation Identifier Names and Codes; LOINC). This study, which is exempt from informed consent requirements, involves secondary analysis of pre-existing, de-identified data. The de-identification process complies with the standards set forth in Section §164.514(a) of the Health Insurance Portability and Accountability Act (HIPAA) Privacy Rule and has been validated by a qualified expert as specified in Section §164.514(b) [16]. The study received approval from the Institutional Review Board (IRB) of Chung Shan Medical University Hospital (IRB number: CS2-23180), which conforms to the provisions of the Declaration of Helsinki.

### 2.2 Study Participants and Main Outcome

**Figure 1:**
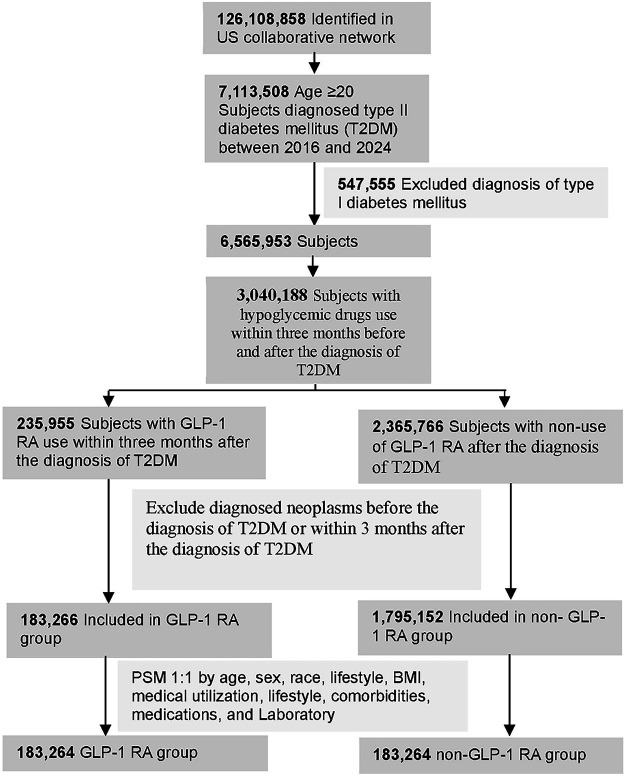
Flowchart illustrating participant selection. T2DM: type 2 diabetes mellitus, GLP-1 RA: glucagon-like peptide-1 receptor agonist, PSM: propensity score matching, BMI: body mass index

Fig. 1 illustrates the flowchart detailing the construction of the study cohort. The participants included adults aged 20 years or older who had been diagnosed withT2DM (ICD-10-CM: E11) between 2016 and 2024. Individuals with a prior diagnosis of type 1 diabetes mellitus (ICD-10-CM: E10) were excluded. To ensure the accuracy of the diagnosis, only subjects who had used hypoglycemic agents within three months before and after the T2DM diagnosis were included.

The exposure group was defined as individuals who had used GLP-1 receptor agonists (GLP-1 RA, ATC: A10BJ) within three months of their T2DM diagnosis, while the non-exposure group consisted of those who had never used GLP-1 RA after their T2DM diagnosis. The index date was defined as the date of initial T2DM diagnosis.

The study outcome was defined as a diagnosis of malignant neoplasms (Detailed codes are provided in Table A1). To ensure the identification of newly diagnosed outcomes, both groups excluded individuals who had been diagnosed with neoplasms (ICD-10-CM: C00–D49) before or within three months after their T2DM diagnosis.

Baseline data were extracted from medical records covering the year preceding the index date up to the day before it. Demographic variables included age, sex, race, lifestyle factors, and body mass index (BMI). Information on healthcare utilization comprised records of outpatient, emergency department, and inpatient visits. Baseline comorbidities assessed included essential (primary) hypertension, overweight and obesity, unspecified hyperlipidemia, ischemic heart disease, chronic kidney disease, cerebrovascular disease, other chronic obstructive pulmonary diseases, liver fibrosis and cirrhosis, and chronic viral hepatitis. Medication use was evaluated for insulins and analogues, biguanides, sulfonylureas, sodium-glucose co-transporter 2 (SGLT2) inhibitors, dipeptidyl peptidase-4 (DPP-4) inhibitors, thiazolidinediones, and alpha-glucosidase inhibitors. Laboratory measurements included hemoglobin A1c (%), leukocyte count (10^3^/μL), C-reactive protein (mg/L), and estimated glomerular filtration rate (eGFR, mL/min/1.73 m^2^) based on a creatinine-based modification of diet in renal disease (MDRD) formula. All relevant diagnostic and procedural codes are listed in Table A2.

To address baseline differences and control for confounding variables, propensity score matching (PSM) was conducted in a 1:1 ratio using TriNetX’s built-in functionality. The matching process accounted for variables including age, sex, race, lifestyle factors, body mass index (BMI), healthcare utilization, comorbidities, medications, and laboratory values. A greedy nearest-neighbor algorithm with a caliper of 0.1 pooled standard deviations was utilized for the matching. The primary aim was to compare cancer risk. Follow-up began 91 days after the index date and continued until cancer occurred or the last available medical record was reached.

### 2.3 Statistical Analysis

Baseline characteristics of the GLP-1 receptor agonist (GLP-1 RA) and non–GLP-1 RA cohorts were assessed using standardized mean differences (SMD), with an SMD <0.1 indicating adequate covariate balance. Continuous variables are reported as means with standard deviation (SD), whereas categorical variables are presented as frequencies and corresponding percentages. Missing values were acknowledged and handled according to the platform’s default procedures. To assess the risk of malignant neoplasms between the GLP-1 receptor agonist (GLP-1 RA) group and the non-GLP-1 RA group, Kaplan–Meier survival curves and Cox proportional hazards models were applied. Stratified analyses were additionally conducted by body mass index (BMI), examining subgroups with BMI < 30 and BMI ≥ 30 to further explore potential differences in cancer risk between the two cohorts. All statistical analyses were performed on the TriNetX platform, which utilizes R version 4.0.2 as its underlying analytic engine.

## 3 Results

### 3.1 Study Cohort and Patient Characteristics

A total of 126,108,858 individuals were identified from the US collaborative network, from which 7,113,508 adults (≥20 years) diagnosed with T2DM between 2016 and 2024 were eligible for inclusion. After exclusion of individuals with prior neoplasms and type 1 diabetes, and after ensuring hypoglycemic medication use around diagnosis, the final analytical sample comprised 235,955 GLP-1 RA users and 2,365,766 non-users. Post 1:1 propensity score matching (PSM) on demographics, lifestyle, comorbidities, medication usage, and laboratory parameters, 183,264 subjects were included in both the GLP-1 RA and non-GLP-1 RA groups. Baseline characteristics were well balanced post-PSM, with all standardized mean differences (SMD) <0.1. The mean age was 55.7 years in both groups, with comparable sex, race, BMI categories, and clinical covariates. Prior to matching, the GLP-1 RA group had higher BMI and greater ambulatory healthcare utilization, and a lower prevalence of ischemic heart diseases, chronic kidney disease, cerebrovascular diseases, and chronic obstructive pulmonary disease; these differences were attenuated post-matching (Table 1).

**Table 1:** Demographic characteristics of Glucagon-like peptide-1 receptor agonist (GLP-1 RA) and non-GLP-1 RA

Variable	Before PSM			After PSM		
	GLP-1 RA	Non-GLP-1 RA	SMD	GLP-1 RA	Non-GLP-1 RA	SMD
	N = 183,266	N = 1,795,152		N = 183,264	N = 183,264	
**Age at index (year), Mean ± SD**	55.71 ± 12.85	60.80 ± 13.62	0.384	55.71 ± 12.85	55.78 ± 14.45	0.005
**Sex**						
Female, (N, %)	98,516 (53.76)	794,373 (44.25)	0.191	98,514 (53.76)	97,598 (53.26)	0.010
Male, (N, %)	84,451 (46.08)	952,506 (53.06)	0.140	84,451 (46.08)	85,280 (46.53)	0.009
**Race**						
White, (N, %)	113,175 (61.76)	1,026,183 (57.16)	0.094	113,174 (61.76)	113,636 (62.01)	0.005
Black or African American, (N, %)	33,162 (18.10)	318,388 (17.74)	0.009	33,161 (18.10)	32,656 (17.82)	0.007
Asian, (N, %)	6763 (3.69)	100,153 (5.58)	0.090	6763 (3.69)	6707 (3.66)	0.002
Other race, (N, %)	11,344 (6.19)	105,017 (5.85)	0.014	11,344 (6.19)	11,611 (6.34)	0.006
Unknown race, (N, %)	16,340 (8.92)	216,891 (12.08)	0.103	16,340 (8.92)	16,247 (8.87)	0.002
**Lifestyle**						
Personal history of nicotine dependence, (N, %)	9518 (5.19)	185,606 (10.34)	0.193	9518 (5.19)	9491 (5.18)	0.001
Tobacco use, (N, %)	2788 (1.52)	38,628 (2.15)	0.047	2788 (1.52)	2710 (1.48)	0.004
Persons with potential health hazards related to socioeconomic, (N, %) and psychosocial circumstances	1667 (0.91)	35,656 (1.99)	0.090	1667 (0.91)	1619 (0.88)	0.003
Nicotine dependence, (N, %)	10,642 (5.81)	203,466 (11.33)	0.198	10,642 (5.81)	10,430 (5.69)	0.005
Alcohol related disorders, (N, %)	1610 (0.88)	54,079 (3.01)	0.155	1610 (0.88)	1604 (0.88)	<0.001
**BMI, Mean ± SD**	36.30 ± 8.44	31.89 ± 8.11	0.533	36.30 ± 8.44	35.61 ± 8.34	0.082
<30, (N, %)	29,708 (16.21)	501,879 (27.96)	0.286	29,708 (16.21)	29,616 (16.16)	0.001
30–34, (N, %)	35,873 (19.57)	336,489 (18.74)	0.021	35,873 (19.57)	36,040 (19.67)	0.002
≥35, (N, %)	64,224 (35.04)	353,430 (19.69)	0.350	64,222 (35.04)	63,015 (34.39)	0.014
**Medical Utilization**						
Ambulatory, (N, %)	128,920 (70.35)	1,015,390 (56.56)	0.289	128,919 (70.35)	128,639 (70.19)	0.003
Emergency, (N, %)	22,817 (12.45)	410,076 (22.84)	0.275	22,817 (12.45)	22,240 (12.14)	0.010
Inpatient encounter, (N, %)	20,446 (11.16)	604,893 (33.70)	0.561	20,446 (11.16)	20,331 (11.09)	0.002
**Comorbidities**						
Essential (primary) hypertension, (N, %)	98,001 (53.48)	1,014,408 (56.51)	0.061	98,000 (53.48)	97,712 (53.32)	0.003
Overweight and obesity, (N, %)	49,793 (27.17)	331,152 (18.45)	0.209	49,791 (27.17)	48,348 (26.38)	0.018
Hyperlipidemia, unspecified, (N, %)	53,874 (29.40)	608,600 (33.90)	0.097	53,873 (29.40)	53,342 (29.11)	0.006
Ischemic heart diseases, (N, %)	21,495 (11.73)	386,933 (21.55)	0.266	21,495 (11.73)	21,381 (11.67)	0.002
Chronic kidney disease, (N, %)	13,943 (7.61)	255,116 (14.21)	0.213	13,943 (7.61)	13,903 (7.59)	0.001
Cerebrovascular diseases, (N, %)	7211 (3.94)	162,154 (9.03)	0.208	7211 (3.94)	7395 (4.04)	0.005
Other chronic obstructive pulmonary disease, (N, %)	6321 (3.45)	150,065 (8.36)	0.209	6321 (3.45)	6341 (3.46)	0.001
Fibrosis and cirrhosis of liver, (N, %)	1679 (0.92)	31,531 (1.76)	0.073	1679 (0.92)	1651 (0.90)	0.002
Chronic viral hepatitis, (N, %)	458 (0.25)	11,563 (0.64)	0.059	458 (0.25)	497 (0.27)	0.004
**Medications**						
Insulins and analogues, (N, %)	52,730 (28.77)	671,481 (37.41)	0.184	52,730 (28.77)	52,613 (28.71)	0.001
Biguanides, (N, %)	78,596 (42.89)	733,510 (40.86)	0.041	78,596 (42.89)	78,897 (43.05)	0.003
Sulfonylureas, (N, %)	25,582 (13.96)	225,391 (12.56)	0.041	25,581 (13.96)	25,799 (14.08)	0.003
Sodium-glucose co-transporter 2 (SGLT2) inhibitors, (N, %)	27,900 (15.22)	94,932 (5.29)	0.332	27,898 (15.22)	28,197 (15.39)	0.005
Dipeptidyl peptidase 4 (DPP-4) inhibitors, (N, %)	11,137 (6.08)	111,561 (6.22)	0.006	11,136 (6.08)	11,315 (6.17)	0.004
Thiazolidinediones, (N, %)	7167 (3.91)	36,675 (2.04)	0.110	7166 (3.91)	7167 (3.91)	<0.001
Alpha glucosidase inhibitors, (N, %)	379 (0.21)	2390 (0.13)	0.018	379 (0.21)	360 (0.20)	0.002
**Laboratory**						
Hemoglobin A1c (%), Mean ± SD	8.17 ± 2.29	7.79 ± 2.31	0.165	8.17 ± 2.29	7.86 ± 2.21	0.136
Leukocytes (10^3^/μL), Mean ± SD	9.32 ± 58.09	10.53 ± 72.57	0.018	9.32 ± 58.09	10.01 ± 72.42	0.010
C reactive protein (mg/L), Mean ± SD	38.32 ± 68.80	61.96 ± 84.42	0.307	38.32 ± 68.80	50.16 ± 76.69	0.162
Glomerular filtration rate/1.73 sq M.predicted [Volume Rate/Area] in Serum, Plasma or Blood by Creatinine-based formula (Modification of diet in renal disease), Mean ± SD	80.01 ± 29.32	72.13 ± 33.56	0.250	80.01 ± 29.32	80.02 ± 32.02	<0.001

Note: SMD: standardized mean difference, GLP-1 RA: glucagon-like peptide-1 receptor agonist, PSM: propensity score matching, SD: standard deviation, BMI: body mass index.

### 3.2 Outcome: Risk of All Malignant Neoplasms

**Figure 2:**
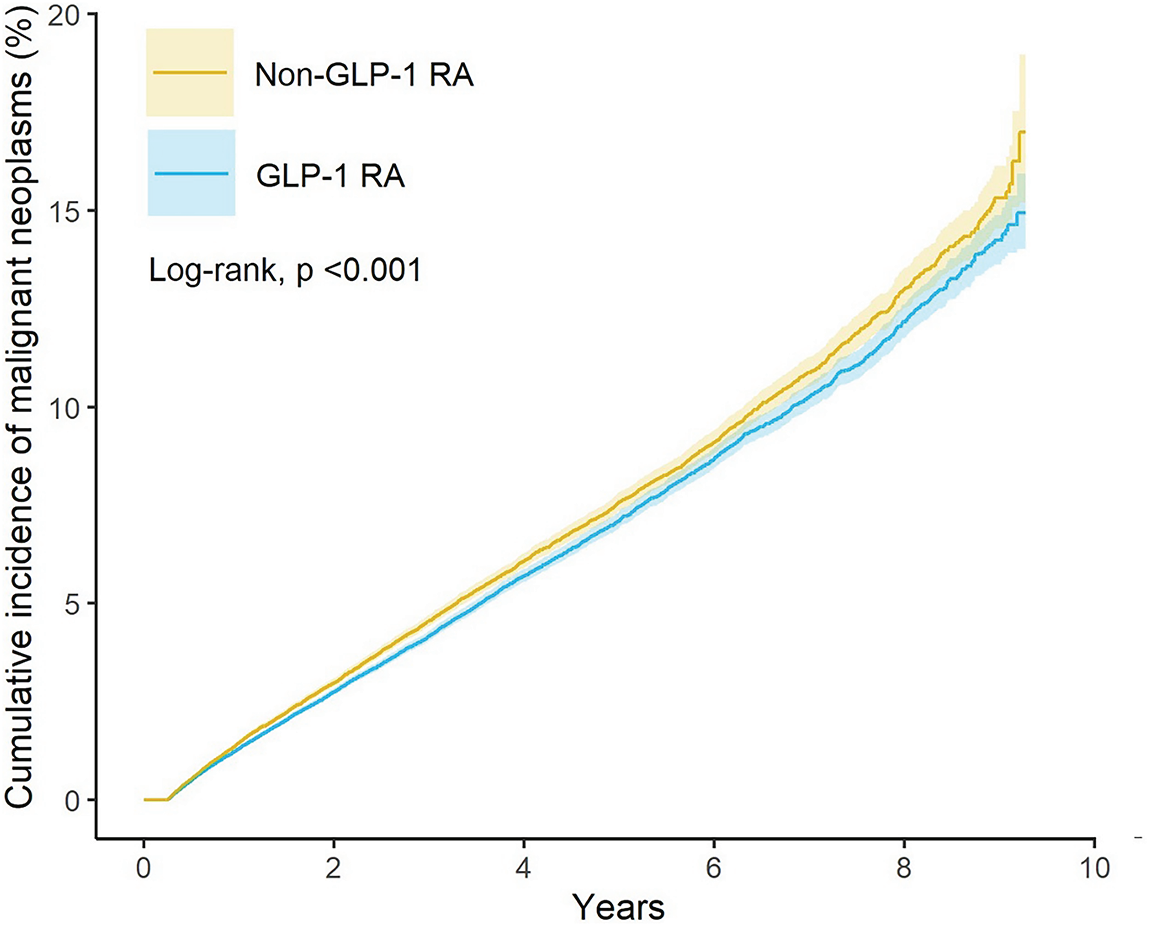
Kaplan-Meier curves showing the risk of all malignant neoplasms over time in GLP-1 RA users and matched non-users

**Figure 3:**
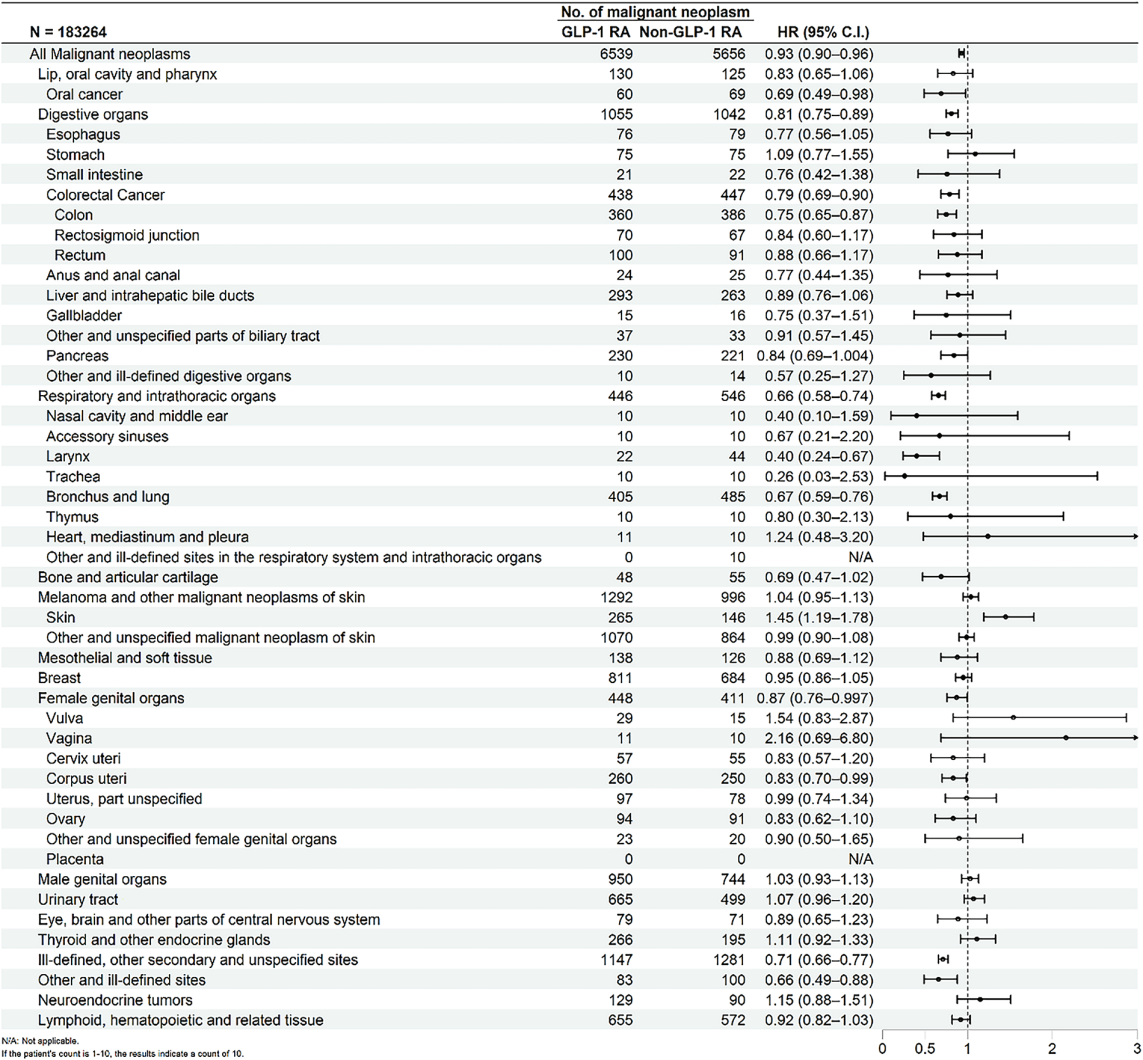
Forest plot comparing the risk of malignant neoplasms between GLP-1 RA users and non-users

Fig. 2 presented the Kaplan-Meier curves depicting cumulative incidence of all malignant neoplasms over time between GLP-1 RA users and matched non-users. Log-rank testing confirmed that the difference in cumulative incidence of all malignant neoplasms was statistically significant. Fig. 3 presented the risk of malignant neoplasms between GLP-1 RA and non-GLP-1 RA groups, demonstrating a hazard ratio (HR) of 0.93 (95% CI: 0.90–0.96), which indicates a statistically significant reduction in the overall risk of cancer. Site-specific analyses revealed notably lower risks for cancers of the digestive system (HR: 0.81; 95% CI: 0.75–0.89), including colon cancer (HR: 0.75; 95% CI: 0.65–0.87), and for malignancies of the respiratory tract (HR: 0.66; 95% CI: 0.58–0.74), such as laryngeal cancer (HR: 0.40; 95% CI: 0.24–0.67) and bronchus and lung cancer (HR: 0.67; 95% CI: 0.59–0.76). Additional reductions in cancer risk were observed for malignancies of the female genital organs (HR: 0.87; 95% CI: 0.76–0.997) and oral cancer (HR: 0.69; 95% CI: 0.49–0.98). In contrast, GLP-1 RA use was associated with a significantly increased risk of malignant melanoma (HR: 1.45; 95% CI: 1.19–1.78).

### 3.3 Outcome: Stratified Analysis of Malignant Neoplasms

Table 2 presented the stratified analysis of cancer risk according to body mass index (BMI). Among individuals with BMI ≥ 30, GLP-1 RA use was associated with a significantly lower risk of overall malignancy (HR: 0.91; 95% CI: 0.87–0.96). Notable reductions were observed for cancers of the digestive system (HR: 0.82; 95% CI: 0.72–0.93), including pancreatic cancer (HR: 0.74; 95% CI: 0.56–0.99) and colorectal cancer (HR: 0.78; 95% CI: 0.64–0.96), as well as for respiratory tract cancers (HR: 0.72; 95% CI: 0.59–0.88). In contrast, among individuals with BMI < 30, no significant reduction in the overall risk of malignancy was observed (HR: 0.97; 95% CI: 0.89–1.05). However, a protective association remained evident for bronchus and lung cancer (HR: 0.71; 95% CI: 0.53–0.95).

**Table 2:** Risk of Malignant neoplasms between GLP-1 RA and non-GLP-1 RA by BMI

Category	BMI < 30 (No. of Event)			BMI ≥ 30 (No. of Event)		
	GLP-1 RA	Non-GLP-1 RA	HR (95% C.I.)	GLP-1 RA	Non-GLP-1 RA	HR (95% C.I.)
	N = 29,400	N = 29,400		N = 91,585	N = 91,585	
All Malignant neoplasms, (N)	1110	954	0.97 (0.89–1.05)	2932	2582	0.91 (0.87–0.96)
Lip, oral cavity and pharynx, (N)	19	18	0.86 (0.45–1.64)	64	58	0.89 (0.62–1.26)
Oral cancer, (N)	10	12	0.54 (0.22–1.33)	29	33	0.71 (0.43–1.17)
Digestive organs, (N)	211	161	1.08 (0.88–1.33)	465	457	0.82 (0.72–0.93)
Esophagus, (N)	14	10	1.66 (0.67–4.10)	43	32	1.08 (0.69–1.71)
Stomach, (N)	17	17	1.77 (0.76–4.10)	33	33	0.88 (0.54–1.45)
Small intestine, (N)	10	10	0.83 (0.21–3.32)	11	11	0.80 (0.35–1.86)
Colorectal Cancer, (N)	75	62	1.00 (0.71–1.40)	192	197	0.78 (0.64–0.96)
Colon, (N)	64	55	0.96 (0.67–1.38)	156	177	0.71 (0.57–0.88)
Rectosigmoid junction, (N)	11	14	0.65 (0.30–1.43)	33	37	0.71 (0.45–1.14)
Rectum, (N)	16	14	0.95 (0.46–1.95)	42	26	1.29 (0.79–2.11)
Anus and anal canal, (N)	10	10	1.24 (0.35–4.39)	12	10	1.20 (0.49–2.93)
Liver and intrahepatic bile ducts, (N)	62	35	1.46 (0.97–2.21)	124	116	0.86 (0.67–1.11)
Gallbladder, (N)	10	10	0.27 (0.03–2.64)	10	10	0.71 (0.26–1.95)
Other and unspecified parts of biliary tract, (N)	10	10	0.52 (0.17–1.58)	18	15	0.98 (0.49–1.94)
Pancreas, (N)	56	43	1.08 (0.73–1.61)	92	99	0.74 (0.56–0.99)
Other and ill-defined digestive organs, (N)	10	10	0.42 (0.08–2.27)	10	10	0.55 (0.21–1.45)
Respiratory and intrathoracic organs, (N)	86	100	0.71 (0.53–0.95)	176	196	0.72 (0.59–0.88)
Nasal cavity and middle ear, (N)	0	10	N/A	10	10	0.79 (0.11–5.60)
Accessory sinuses, (N)	10	0	N/A	10	10	1.21 (0.20–7.21)
Larynx, (N)	10	10	0.55 (0.09–3.31)	10	22	0.26 (0.11–0.60)
Trachea, (N)	0	0	N/A	0	10	N/A
Bronchus and lung, (N)	81	95	0.71 (0.53–0.95)	162	170	0.76 (0.61–0.95)
Thymus, (N)	10	10	0.85 (0.05–13.61)	10	10	0.38 (0.04–4.23)
Heart, mediastinum and pleura, (N)	10	10	0.40 (0.04–4.42)	10	10	0.65 (0.20–2.13)
Other and ill-defined sites in the respiratory, (N) system and intrathoracic organs, (N)	0	10	N/A	0	10	N/A
Bone and articular cartilage, (N)	10	10	0.84 (0.24–2.90)	20	17	0.94 (0.49–1.79)
Melanoma and other malignant neoplasms of skin, (N)	210	169	1.03 (0.84–1.26)	525	399	1.05 (0.93–1.20)
Malignant melanoma of skin, (N)	33	23	1.18 (0.70–2.02)	119	80	1.19 (0.90–1.58)
Other and unspecified malignant neoplasm of skin, (N)	176	152	0.96 (0.77–1.19)	432	326	1.06 (0.92–1.23)
Mesothelial and soft tissue, (N)	18	25	0.60 (0.33–1.09)	70	58	0.96 (0.68–1.36)
Breast, (N)	138	109	1.03 (0.80–1.32)	409	334	0.99 (0.85–1.14)
Female genital organs, (N)	43	31	1.12 (0.70–1.77)	257	242	0.85 (0.72–1.02)
Vulva, (N)	10	0	N/A	15	10	2.00 (0.78–5.16)
Vagina, (N)	10	10	1.61 (0.15–17.77)	10	10	1.00 (0.27–3.71)
Cervix uteri, (N)	10	10	1.34 (0.49–3.70)	21	33	0.51 (0.30–0.88)
Corpus uteri, (N)	19	15	1.02 (0.52–2.00)	155	161	0.77 (0.62–0.96)
Uterus, part unspecified, (N)	10	10	0.92 (0.33–2.53)	53	52	0.81 (0.55–1.19)
Ovary, (N)	10	10	0.91 (0.35–2.35)	58	53	0.88 (0.60–1.27)
Other and unspecified female genital organs, (N)	10	10	0.79 (0.05–12.68)	14	14	0.79 (0.38–1.65)
Placenta, (N)	0	10	N/A	0	0	N/A
Male genital organs, (N)	198	140	1.17 (0.94–1.45)	424	342	1.01 (0.88–1.16)
Urinary tract, (N)	115	75	1.28 (0.95–1.71)	264	247	0.86 (0.73–1.03)
Eye, brain and other parts of central nervous system, (N)	13	14	0.77 (0.36–1.63)	44	36	0.99 (0.64–1.53)
Thyroid and other endocrine glands, (N)	37	31	1.00 (0.62–1.60)	129	127	0.83 (0.65–1.06)
Ill-defined, other secondary and unspecified sites, (N)	207	203	0.84 (0.69–1.02)	510	553	0.74 (0.65–0.83)
Other and ill-defined sites, (N)	18	15	1.00 (0.50–1.98)	34	48	0.56 (0.36–0.87)
Neuroendocrine tumors, (N)	17	15	0.94 (0.47–1.89)	60	42	1.14 (0.77–1.70)
Lymphoid, hematopoietic and related tissue, (N)	105	100	0.87 (0.66–1.14)	300	277	0.87 (0.74–1.03)

Note: If the patient’s count is 1–10, the results indicate a count of 10. N/A: Not applicable, HR: hazard ratio, CI: confidence interval.

## 4 Discussion

As GLP-1 RA therapy has been adopted progressively earlier in the T2DM disease course, understanding its long-term safety—particularly oncologic outcomes—has become increasingly important. Previous evidence indicates that GLP-1 RA therapy was typically initiated a median of 6.1 years after T2DM diagnosis, reflecting delayed adoption in clinical practice [17]. However, growing evidence suggests that earlier initiation may lead to better glycemic outcomes and more durable long-term control [18,19]. Despite these metabolic benefits, the potential impact of early GLP-1 RA use on cancer risk remains unclear and warrants further investigation. This historical delay in GLP-1 RA initiation—often several years post-diagnosis—introduces potential confounding, as interim exposure to other antidiabetic agents may influence cancer risk. Consequently, isolating the specific effects of GLP-1 RA has been challenging. Our study aimed to specifically evaluate the impact of early initiation—defined as within three months of diagnosis—on subsequent cancer development. This design confers several methodological advantages. First, restricting GLP-1 RA exposure to the early disease stage minimizes exposure misclassification and ensures a consistent temporal sequence between treatment and cancer onset. Second, it helps mitigate reverse causality by reducing the likelihood that preclinical malignancies influenced treatment decisions. Together, these elements strengthen causal inference by upholding the core epidemiologic principle that exposure must precede outcome.

Compared with non-use, GLP-1 RA use was associated with a modest but statistically significant reduction in overall cancer incidence (hazard ratio [HR], 0.93; 95% confidence interval [CI], 0.90–0.97). Subgroup analyses by cancer type revealed decreased risk for several malignancies, including cancers of the respiratory tract (HR, 0.63; 95% CI, 0.45–0.89), digestive organs (HR, 0.80; 95% CI, 0.72–0.89), and colorectum (HR, 0.79; 95% CI, 0.66–0.96). The risk of pancreatic cancer was also modestly lower among GLP-1 RA users (HR, 0.84; 95% CI, 0.69–1.00), although the upper bound of the confidence interval approached unity. In contrast, GLP-1 RA use was associated with an increased risk of malignant melanoma (HR, 1.31; 95% CI, 1.01–1.70), while no statistically significant increase was observed for breast cancer (HR, 1.04; 95% CI, 0.94–1.15) or thyroid cancer (HR, 1.09; 95% CI, 0.88–1.34). The reduced risk of colorectal, digestive tract, and respiratory tract cancers among early GLP-1 RA users aligns with findings from several recent population-based studies [15,20,21]. Earlier observational studies had raised concerns about a potential increase in site-specific cancers—particularly thyroid, pancreatic, and breast malignancies—associated with GLP-1 RA exposure [10,22,23]. However, more recent large-scale cohort studies have not substantiated these associations, instead reporting neutral or null findings [12,24]. Our results support these more contemporary observations, showing no significant association in the risk of thyroid or breast cancer among early GLP-1 RA users.

The elevated risk of malignant melanoma observed in our cohort warrants cautious interpretation. To date, this association has not been consistently reported in the literature, and no well-established biological mechanism has been proposed. A recent population-based cohort study found no significant increase in the risk of melanoma (HR, 0.96; 95% CI, 0.53–1.75) or non-melanoma skin cancers (HR, 1.03; 95% CI, 0.80–1.33) among GLP-1 RA users compared to sulfonylurea users [25]. This discrepancy may reflect differences in population characteristics, confounding control, or outcome ascertainment. Further research is needed to validate our findings and determine whether the observed signal reflects a true drug-related effect or residual bias. To further explore the heterogeneity of cancer risk reduction associated with GLP-1 RA use, we performed a stratified analysis based on BMI. Notably, the risk reduction in overall cancer incidence was more pronounced among individuals with BMI ≥ 30 (HR, 0.91; 95% CI, 0.87–0.96), compared to those with BMI < 30 (HR, 0.97; 95% CI, 0.89–1.05).

While our findings are broadly consistent with a recent nationwide analysis of GLP-1 RA use and cancer risk in individuals with obesity [26], our study provides additional insights by focusing on a clearly defined early exposure period within a diabetes-specific population. In contrast to the prior study, which enrolled obese individuals regardless of glycemic status, our analysis was limited to patients with confirmed T2DM, allowing for a more targeted assessment within the population for whom GLP-1 RA are primarily indicated. Similarly, a study by Wang et al., which focused on overweight and obese drug-naïve patients initiating antidiabetic therapy, found that GLP-1 RA were associated with a 50% lower risk of colorectal cancer compared to insulin, and a 42% lower risk compared to metformin during a median follow-up of 3 years [15]. Site-specific analyses showed a significant reduction in digestive organ cancers (HR, 0.82; 95% CI, 0.72–0.93) and pancreatic cancer (HR, 0.74; 95% CI, 0.56–0.99) in the higher BMI subgroup, whereas no such benefit was observed in the lower BMI group. These findings suggest that the potential antineoplastic effects of GLP-1 RA may be more pronounced in individuals with obesity, potentially reflecting the compounds’ dual metabolic and weight-reducing properties.

Several biological mechanisms may provide plausible explanations for the associations observed in our study. From a mechanistic perspective, GLP-1 RA activates the PI3K/AKT/mTOR signaling cascade, a pathway that is indispensable for pancreatic β-cell survival and glucose homeostasis, yet simultaneously represents a canonical oncogenic axis [3]. This dual role highlights the necessity of carefully evaluating the long-term oncologic safety of GLP-1 RA in clinical populations.

Emerging evidence further indicates that GLP-1 RA can modulate the gut microbiota, promoting the expansion of beneficial genera [27,28]. As dysbiosis of the gut microbiome has been implicated in chronic inflammation and tumorigenesis, these observations suggest that microbiota–immune interactions may represent an additional pathway through which GLP-1 RA influences cancer risk.

Furthermore, GLP-1 RA exhibit immunomodulatory properties, including the suppression of nuclear factor kappa-light-chain-enhancer of activated B cells (NF-κB) activation, reductions in pro-inflammatory cytokines such as tumor necrosis factor-alpha (TNF-α) and IL-6, and the promotion of IL-10 secretion through T cell and microglial regulation [29,30]. Together, these pleiotropic effects provide a biologically plausible framework linking GLP-1 RA to cancer-related outcomes. Diabetes is increasingly recognized as a chronic inflammatory condition. A recent meta-analysis identified the ICAM-1 rs5498 polymorphism as a risk factor for T2DM, particularly in Asian populations [31], underscoring the role of immune–inflammatory pathways in diabetes pathogenesis.

In addition to these general mechanisms, the more pronounced cancer risk reduction observed in individuals with obesity may be explained by several factors. First, patients with elevated BMI inherently carry a higher baseline risk for various malignancies, including colorectal, pancreatic, and liver cancers [32,33]. Recent evidence demonstrates that obesity impairs CD8^+^ T cell function within the tumor microenvironment, leading to compromised immune surveillance and inefficient immunoediting, thereby increasing susceptibility to malignancy [34]. Key pathways linking obesity and cancer include PI3K/Akt/mTOR and JAK/STAT3 activation. More recent concepts further implicate the kynurenine–aryl hydrocarbon receptor axis and serine protease–mediated degradation of dependence receptors such as deleted in colorectal cancer and neogenin as additional routes promoting carcinogenesis [35]. Second, beyond glycemic control, GLP-1 RA exert metabolic and anti-inflammatory effects—such as weight reduction, improved insulin sensitivity, and lowered systemic inflammation—that are particularly relevant in the context of obesity [36]. Lastly, obese individuals may exhibit greater physiological responsiveness to GLP-1 RA, achieving more substantial reductions in body weight and metabolic burden, which could synergistically enhance the compounds’ potential antineoplastic effects.

Beyond clinical implications, our findings should be viewed within the broader context of medical innovation and health system resilience. The concept of health system resilience has been increasingly elaborated in recent literature, highlighting how governance, adaptability, and institutional networks enable systems to withstand shocks and sustain essential services under stress [37–39]. In parallel, the rapid adoption of GLP-1 RA illustrates how novel therapies can shape both patient outcomes and healthcare sustainability.

At the individual level, the heterogeneity of cancer risk reduction—particularly the more pronounced effects observed in patients with obesity—underscores the importance of precision medicine. Potential biomarkers such as BMI, systemic inflammatory markers, genetic variations (e.g., ICAM-1 polymorphisms), and gut microbiota composition could inform stratification strategies in future studies. Incorporating these factors into trial design and clinical practice may enable more individualized risk–benefit assessments, ensuring that GLP-1 RA are deployed not only for metabolic control but also as part of tailored strategies to mitigate diabetes-associated cancer risk.

Diverse limitations should be acknowledged in the interpretation of these results. First, although propensity score matching was applied to balance baseline characteristics, some covariates (e.g., HbA1c [SMD 0.136] and CRP [SMD 0.162]) remained slightly imbalanced. In addition, residual confounding from unmeasured variables—such as family history of cancer, lifestyle factors (including diet and physical activity), and environmental exposures—cannot be excluded. Second, the TriNetX platform lacks detailed information on medication adherence, dosage, and duration, limiting the ability to assess dose-response relationships or the effects of long-term GLP-1 RA exposure. Third, cancer outcomes were identified using ICD-10 codes, which may be subject to misclassification or underreporting, particularly for early-stage or subclinical cancers. Fourth, because the data were derived from U.S.-based health care institutions within the TriNetX network, the generalizability of these findings to other populations or health systems may be limited. Finally, as an observational study, causal inference cannot be established. The observed associations between GLP-1 RA use and cancer risk may be influenced by indication bias or other clinical decision-making factors not captured in the database.

## 5 Conclusion

In conclusion, early initiation of GLP-1 receptor agonist was associated with a reduced overall cancer risk, particularly for digestive, respiratory, and female-genital malignancies. These agents also provide significant weight-loss benefits, which may contribute to their protective effect. The risk reduction was especially evident in individuals with obesity, highlighting the dual metabolic and oncologic advantages of timely treatment.

## Data Availability

Research data supporting this publication can be accessed through the TriNetX platform, available at https://trinetx.com/. Data sharing is not applicable to this article, as no datasets were generated or analyzed during the current study.

## Abbreviations

BMI: Body mass index
GLP-1 RA: Glucagon-like peptide-1 receptor agonist
HR: Hazard ratio
PSM: Propensity score matching
SGLT2: Sodium-glucose co-transporter 2
SMD: Standardized mean differences
T2DM: Type 2 diabetes

## Appendix A

**Table A1:** Diagnostic codes for malignant neoplasms

Content	ICD-10-CM
All malignant neoplasms	C00–C96
Lip, oral cavity and pharynx	C00–C14
Oral cancer	C00–C06
Digestive organs	C15–C26
Malignant neoplasm of esophagus	C15
Malignant neoplasm of stomach	C16
Malignant neoplasm of small intestine	C17
Colorectal cancer	C18–C20
Malignant neoplasm of colon	C18
Malignant neoplasm of rectosigmoid junction	C19
Malignant neoplasm of rectum	C20
Malignant neoplasm of anus and anal canal	C21
Malignant neoplasm of liver and intrahepatic bile ducts	C22
Malignant neoplasm of gallbladder	C23
Malignant neoplasm of other and unspecified parts of biliary tract	C24
Malignant neoplasm of pancreas	C25
Malignant neoplasm of other and ill-defined digestive organs	C26
Respiratory and intrathoracic organs	C30–C39
Malignant neoplasm of nasal cavity and middle ear	C30
Malignant neoplasm of accessory sinuses	C31
Malignant neoplasm of larynx	C32
Malignant neoplasm of trachea	C33
Malignant neoplasm of bronchus and lung	C34
Malignant neoplasm of thymus	C37
Malignant neoplasm of heart, mediastinum and pleura	C38
Malignant neoplasm of other and ill-defined sites in the respiratory system and intrathoracic organs	C39
Bone and articular cartilage	C40–C41
Melanoma and other malignant neoplasms of skin	C43–C44
Malignant melanoma of skin	C43
Other and unspecified malignant neoplasm of skin	C44
Mesothelial and soft tissue	C45–C49
Breast	C50–C50
Female genital organs	C51–C58
Malignant neoplasm of Vulva	C51
Malignant neoplasm of Vagina	C52
Malignant neoplasm of Cervix uteri	C53
Malignant neoplasm of Corpus uteri	C54
Malignant neoplasm of Uterus, part unspecified	C55
Malignant neoplasm of Ovary	C56
Malignant neoplasm of Other and unspecified female genital organs	C57
Malignant neoplasm of Placenta	C58
Male genital organs	C60–C63
Urinary tract	C64–C68
Eye, brain and other parts of central nervous system	C69–C72
Thyroid and other endocrine glands	C73–C75
Ill-defined, other secondary and unspecified sites	C76–C80
Other and ill-defined sites	C76
Neuroendocrine tumors	C7A–C7A
Lymphoid, hematopoietic and related tissue	C81–C96

Note: ICD-10-CM: International Classification of Diseases, Tenth Revision, Clinical Modification. ATC: Anatomical Therapeutic Chemical. TNX: TrinetX curated.

**Table A2:** Code for covariates

Content	ICD-10-CM
**Lifestyle**	
Personal history of nicotine dependence	Z87.891
Tobacco use	Z72.0
Persons with potential health hazards related to socioeconomic and psychosocial circumstances	Z55–Z65
Nicotine dependence	F17.2
Alcohol related disorders	F10
**Comorbidities**	
Essential (primary) hypertension	I10
Overweight and obesity	E66
Hyperlipidemia, unspecified	E78.5
Ischemic heart diseases	I20–I25
Chronic kidney disease	N18
Cerebrovascular diseases	I60–I69
Chronic obstructive pulmonary disease	J44
Fibrosis and cirrhosis of liver	K74
Chronic viral hepatitis	B18
**Medications**	
Insulins and analogues	ATC: A10A
Biguanides	ATC: A10BA
Sulfonylureas	ATC: A10BB
Sodium-glucose co-transporter 2 (SGLT2) inhibitors	ATC: A10BK
Dipeptidyl peptidase 4 (DPP-4) inhibitors	ATC: A10BH
Thiazolidinediones	ATC: A10BG
Alpha glucosidase inhibitors	ATC: A10BF
**Laboratory**	
Hemoglobin A1c (%)	TNX: 9037
Leukocyte count (10^3^/μL)	TNX: 9015
C-reactive protein (mg/L)	TNX: 9063
Estimated glomerular filtration rate (eGFR, mL/min/1.73 m^2^) based on a creatinine-based modification of diet in renal disease formula	TNX: 8001

Note: ICD-10-CM: International Classification of Diseases, Tenth Revision, Clinical Modification.
